# Reactive intermediate phase cold sintering in strontium titanate†

**DOI:** 10.1039/c8ra03072c

**Published:** 2018-06-04

**Authors:** R. Boston, J. Guo, S. Funahashi, A. L. Baker, I. M. Reaney, C. A. Randall

**Affiliations:** Materials Science and Engineering, University of Sheffield S1 3JD UK r.boston@sheffield.ac.uk; Materials Research Institute and Department of Materials Science and Engineering, The Pennsylvania State University PA16802 USA; Murata Mfg Co LtD Kyoto 617-0832 Japan

## Abstract

Dense (>96% theoretical) strontium titanate ceramics were fabricated at 950 °C (conventional sintering temperature > 1400 °C) using a reactive intermediate phase cold sintering process. An aqueous solution of SrCl_2_ mixed with TiO_2_ nanoparticles was added to SrTiO_3_ powders and pressed at 180 °C to obtain a highly compacted green body. During the post-press heating step at 950 °C, the TiO_2_ and SrCl_2_ create in-filling micro-reactions around each grain resulting in dense (>96%) SrTiO_3_ ceramics. Nano- and micron-sized starting powders were used, demonstrating that this reactive intermediate phase cold sintering route can densify a wide range of starting powder sizes, as it not reliant on an amorphous-to-crystalline precipitation through the terrace ledge kink mechanism, as has been identified repeatedly in previous cold sintering mechanisms. Moreover, this process has the potential to densify a wide variety of functional oxides, as a range of different low-temperature chemical synthesis routes could be used.

## Abstract 2

Strontium titanate ceramics (>96% theoretical) have been fabricated using a reactive intermediate phase cold sintering process with a range of starting-powder sizes.

## Graphical abstract

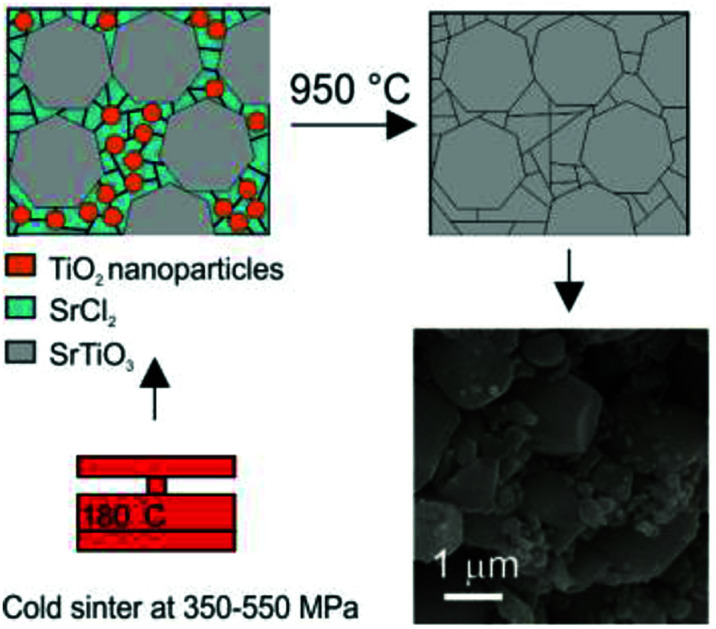

## Introduction

Ceramic technology has existed for thousands of years, initially as a means of fabricating cooking and tableware or ornaments, but more recently in advanced applications for example consumer electronics, energy components, and gas separation and structural applications. Common to these uses has been the need to fire or sinter materials at high temperature (>1000 °C) which drives diffusion processes that enable the powders to undergo densification and grain growth, minimizing the interfacial contributions to the free energy. High temperature processing requires large amounts of energy, leaving ceramics in danger of being left behind in the pursuit of sustainable technologies. Techniques such as spark plasma sintering (SPS) have gone some way to addressing this, and have demonstrated reductions in sintering temperature, in some cases to below 1000 °C, however concerns still remain about carbon contamination and reduction as a result.^1−3^ Recently however, a new, low temperature consolidation technique has been developed, known as the Cold Sintering (CS) process,^4,5^ which has been shown to significantly reduce the sintering temperature of wide range of functional oxides.^4−7^ CS is potentially revolutionary in terms of both energy saving and in the creation of new, previously inaccessible material combinations.^4^ The process works by using a small quantity of a transient solvent (often water) to hydrate or partially dissolve the surface of the starting powders, creating a supersaturated solution around each particulate.^4^ The hydrated powder is then uniaxially pressed and simultaneously heated to between 120–300 °C. The pressing enables the hydrated particles to flow around one another, creating a highly compacted green body. The transient solvent is then driven off by simultaneous pressing and *in situ* heating, precipitating the phase to in-fill the voids between grains to create a fully dense ceramic. For soluble materials, the transient solvent is water, and fully dense ceramics may be formed at temperatures many hundreds of degrees below those which are normally used. This technique works well for materials which congruently dissociate in water (*e.g.* Li_2_MoO_4_)^8^ however for more stable materials, or those which do not show congruent dissolution, a proactive addition of solvochemical solutions must be employed.^9,10^ This has been successfully demonstrated in hydrothermal cold sintering of BaTiO_3_. Here titanium dioxide nanoparticles were added to a saturated aqueous solution of barium hydroxide, and used to form a glassy phase around each BaTiO_3_ particle, which was then precipitated through terrace ledge kink (TLK) growth during the post-press heating step at 950 °C.^9^ The heating step is an inescapable side effect of using the solvent route; although requiring a higher temperature than for the congruent route, 950 °C represents a significant reduction in sintering temperature for un-doped BaTiO_3_ (1350 °C).^11^ This approach is limited, however, as creating the precise conditions to form the hydrothermal reaction are critical, and as yet the approach has only been successful in BaTiO_3_.

Cold sintering, hydrothermal or otherwise, relies on TLK growth which requires nanoscale (<50 nm) reagents, with pristine crystalline surfaces.^9^ Both of these requirements limit the wider applicability of the method due to the difficulties in producing high quality nanopowders, particularly in complex compositions. A new processing route which removes these two requirements is therefore highly desirable for cold sintering of oxides. If micron-sized solid-state powders may be successfully cold sintered, this drastically expands the range of materials to which the technique may be applied (*i.e.* solid state powders).

Recently a new synthesis route for bulk barium titanate powders has been developed, using a malonic acid/choline chloride deep eutectic solvent to control and direct the formation of barium titanate at temperatures lower than the solid state (950 °C).^12^ The method was shown to lower the reaction temperature compared to solid-state processing through the formation of nanoscale BaCl_2_ and TiO_2_ as intermediate phases. The reduction in reaction temperature is driven by the interaction of these two intermediates, which liberates the chloride ions as the product forms. This previous work provides proof of concept that titanate materials can be created at lowered temperatures using a reaction of nanoscale titanium dioxide and a chloride phase. If this chloride/TiO_2_ reaction can be replicated on the nanoscale around particles of product, then this synthesis route could also be used to create fully dense ceramics. The use of chlorides rather than hydroxides greatly increases the range of materials to which CS could be applied, as there is a very wide variety of highly soluble metal chlorides materials available.

Strontium titanate is an important end-member material for a wide variety of applications. Its ABO_3_ structure makes it highly tolerant of dopants,^13^ which enables fine control of the electronic properties. For example, A-site doped strontium titanate has been demonstrated as a high figure of merit n-type thermoelectric material,^14^ with B-site doping of La–Sr–Ti materials producing properties suitable for solid oxide fuel cell anodes.^15^ Doped SrTiO_3_ also has a highly tunable dielectric constant,^16^ making it an important potential material for phased array antennas,^17,18^ varistors,^19^ or device components which work in the microwave range.^20^ Currently, the most common way to sinter SrTiO_3_ is using traditional high temperature methods, with the base material requiring temperatures > 1400 °C to densify to >95% of theoretical.^21^ Whilst effective, this type of high temperature processing has a number of inherent drawbacks such as high energy costs and sometimes uncontrolled grain growth, meaning that any potentially desirable nanostructures (*e.g.* for the control of thermal conductivity in thermoelectric materials)^22^ are lost. A conventional sintering temperature > 1400 °C also means that SrTiO_3_ is incompatible with conventional internal electrode inks including Pt, restricting its application in multilayer devices. Here, we use the understanding of the titanate/chloride synthetic pathway to create dense SrTiO_3_ (>96% theoretical) by creating reactive intermediate phases using a cold sintering/post press heating process. This is entirely distinct from the hydrothermal-like cold sintering processes, due to the micro-reactions which occur during the post-press heating step, being entirely independent of the bulk powder. We demonstrate that a TiO_2_/chloride solution can be used to create fully dense ceramics from both micron- and nano-sized particles using compaction and recrystallisation of intermediates at 180 °C under uniaxial pressure, followed by heating at 950 °C which promotes reaction of the in-filling intermediate phases creating single-phase, dense ceramics.

## Experimental

All chemicals were obtained from Sigma Aldrich, UK and used without further purification. To pinpoint the required post-cold sintering reaction temperature, SrTiO_3_ was synthesized using the deep eutectic solvent technique described previously^12^ using strontium acetate and titanium isopropoxide precursors. Micron-scale SrTiO_3_ powders were synthesized from SrCO_3_ and TiO_2_ powders using standard solid-state procedures. The particle size was measured to be 1.5 ± 0.5 μm from SEM (Fig. S1a†). Nanoscale SrTiO_3_ and TiO_2_ were obtained and used as received (crystallite size < 100 nm, and < 25 nm respectively) with particle sizes confirmed in Fig. S1b–d.† XRD of the SrTiO_3_ and TiO_2_ powders as produced/received are given in Fig. S2.†

A 1.5 M aqueous solution of SrCl_2_ was created, and 1.5 M equivalent of anatase TiO_2_ nanoparticles (<100 nm) added. Approximately 0.2 ml (which equates to ≈18% equivalent as SrTiO_3_ once reacted) of the solution was added to the chosen powder and ground by hand in a pestle and mortar until it appeared dry, returning to a free-flowing powder. The powder was loaded into a uniaxial hydraulic press die (10 mm diameter) and pressed at an initial pressure of 750 MPa at room temperature for 10 minutes to allow for rearrangement and compaction of the particles inside the die. The temperature of the die was then increased to 180 °C whilst still under load at a heating rate of 20 °C min^−1^. The die was held at temperature for 60 minutes and allowed to cool under load. The applied load was observed to drop by 25–50% during heating. This is due to sample shrinkage caused by particle rearrangement within the die. The green body was then removed from the die and heated in air for 4 h at 950 °C with a heating/cooling rate of 5 °C min^−1^.

Densities of the sintered ceramics were calculated using an Archimedes density balance. X-ray diffraction (XRD) patterns were obtained for all samples using a PANalytical Xpert^3^. Samples were prepared for scanning electron microscopy (SEM) by polishing the ceramics, and by fracturing to examine the internal structure. Samples were mounted on carbon tape and sputtered with a 15 nm thick gold layer as a conductive coating. SEM was performed using a Phillips Inspect F in secondary and backscattered modes, and energy dispersive X-ray analysis (EDX) was conducted using an EDAX EDX detector.

Samples for room temperature relative permittivity were polished and coated with gold paste electrodes before measurements were taken using an LCR meter (Model 4284A, Hewlett Packard, HP).

## Results and discussion

Strontium titanate was successfully synthesized using the deep eutectic solvent method, following the same chemical pathway as the previously reported barium titanate synthesis (See Fig. S3†).^12^ The intermediate phases formed in the reaction indicate that strontium titanate can be formed from well-mixed nanoscale strontium chloride and titanium dioxide at 950 °C. We propose therefore that the reaction detailed here may therefore be used as an intermediate phase reactive cold sintering process to create micro-reactions around each grain to fully densify the ceramic upon heating.

**Fig. 1:**
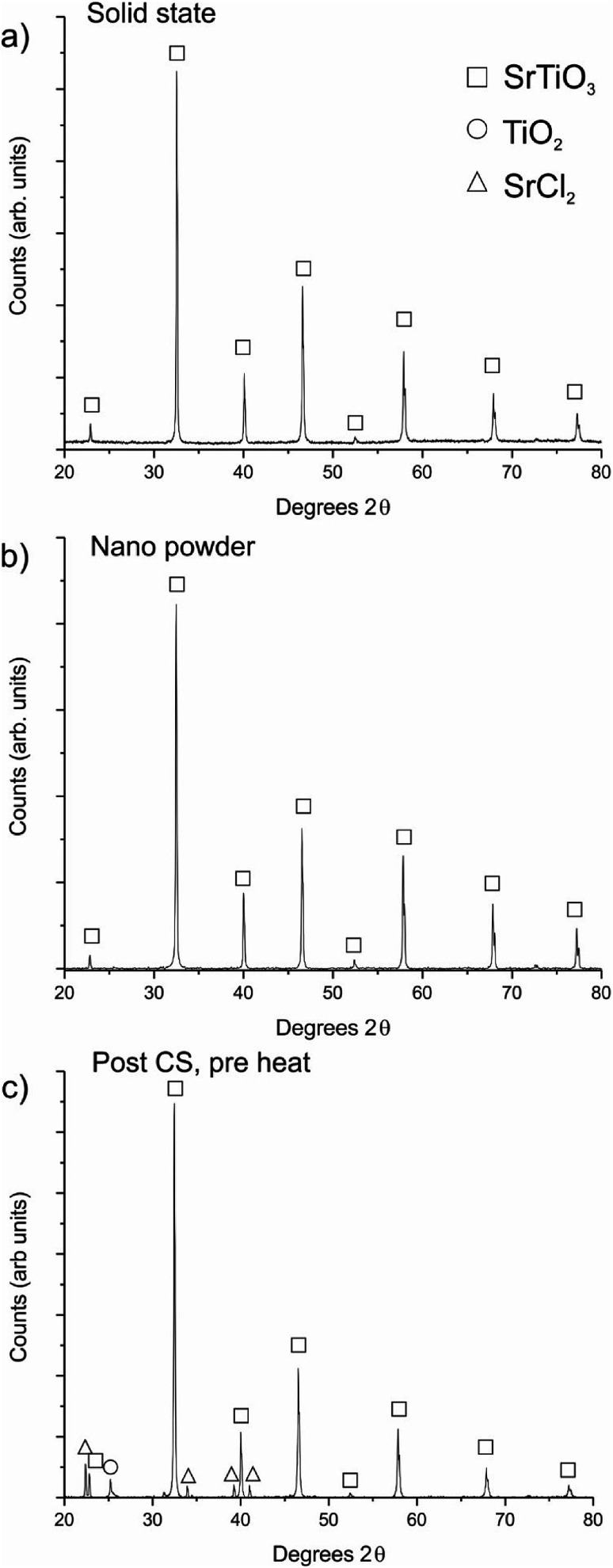
XRD patterns of (a) a CS ceramic created using the solid state powder; (b) a CS ceramic created using the nanopowder; both ceramics are phase pure after the post-press heating step; and (c) a cold sintered ceramic made using the solid state powder before post-press heating, indicating the presence of both the cold sintering solution additives as crystalline phases.

After cold sintering at 180 °C and heating in air at 950 °C, the average densities of the ceramics produced were 97.2 ± 0.8% and 96.2 ± 2.2% of theoretical maximum for the solid state (micron-sized) and nano powders respectively. It should also be noted that, like in other cold sintering processes, there is no lateral shrinkage of the pellets after the post-press heating step, with the ceramics produced having the same diameter before and after the post-press heating stage. XRD was used to determine the phases present in the CS ceramics. Fig. 1a and b shows the XRD patterns for the solid state and nano CS ceramics respectively, indicating that both are single phase strontium titanate. Fig. 1c shows the XRD pattern for a sample after the heated press step but before heating to 950 °C, demonstrating that strontium chloride recrystallises and the TiO_2_ remains crystalline during the hot-pressing procedure, back-filling the spaces between grains with intermediate phases. This creates the conditions for micro-reactions to occur around each grain to form phase pure strontium titanate upon heating to 950 °C.

Control samples were created to decouple the various steps of the method. Table 1 gives the densities achieved for ceramics fabricated under various conditions. Ceramics were uniaxially pressed without heating or addition of solution, followed by heating at 950 °C. In these cases, neither ceramic (solid state or nanopowder) showed any sign of sintering, and were mechanically unstable. Further ceramics were created with added intermediate phase solution but without the in-press heating step (*i.e.* solution was mixed into the SrTiO_3_, pressed at room temperature and heated at 950 °C). The densities of these control ceramics were found to be 63.2 ± 1.7% for the solid-state powder and 63.1 ± 2.9% for the nanopowder calculated using the dimensions and mass (measurements using the Archimedes method were found to be unreliable due to the high level of open porosity of the ceramics). Although mechanically stable, the low density of these ceramics indicates the importance of the heated pressing step. The heated-pressing step (at the heart of the cold sintering process) enables rearrangement and compaction of the particles due to a mobile hydrated phase, followed by recrystallisation of the added strontium chloride phase (Fig. 1c). This allows the multiphase ceramic to retain the densified state until the post-press heating step. Without the concurrent heating/pressing step this densification is lost upon removal of the applied load, as the green body relaxes. Hence, we conclude that the heated pressing step is vital for the production of dense ceramics by “freezing in” the particle compaction through recrystallisation of the SrCl_2_ phase.

**Table 1:** Densities of ceramics formed at 950 °C using conventional uniaxial pressing, room temperature uniaxial pressing and added solution, and intermediate phase reaction CS

SrTiO_3_ source	% of theoretical density		
	No solution added, RT press	Solution added, RT press	Cold sintered
Solid state powder	No sintering	63.2 ± 1.7	97.1 ± 0.8
Nanopowder	No sintering	63.1 ± 2.9	96.2 ± 2.2

**Fig. 2:**
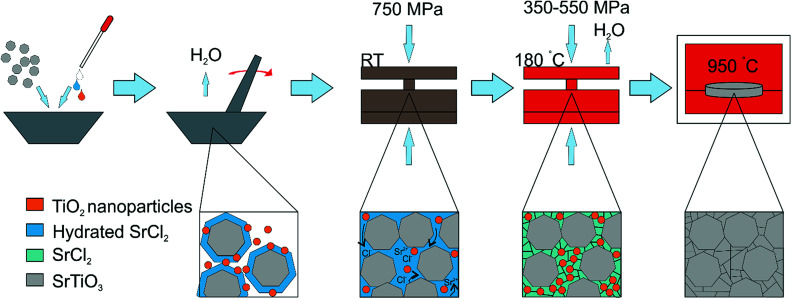
Schematic of the reactive cold sintering process. The pressure falls as the particles rearrange and compact under load. As the temperature is increased there is further densification through removal of the transient liquid phase causing the pellet to further decrease in thickness. Under the additional thermal treatment there is the reactive transformation to a single phase SrTiO_3_ ceramic.

The densification process and the role of each step in intermediate phase reactive CS can therefore be elucidated, as shown in Fig. 2. The infilling aqueous solution hydrates the surface of the strontium titanate powder, which allows flow and rearrangement whilst under applied load at room temperature, creating a ceramic with no voids around the particles as would be seen in standard ceramic pressing. This is observed as a drop in the applied load of approximately 25% as the pellet reduces in height inside the die. When the temperature is increased, the hydrated phases are dried, forming a matrix of crystalline strontium chloride and titanium dioxide around each grain. It is important that this stage is conducted under pressure, as the egress of water would otherwise leave only a partially dense structure, and since the post-press heating temperature is not high enough to promote conventional sintering or grain growth, these small voids would not be refilled. The applied temperature is, however, high enough to melt the SrCl_2_ phase, and it is likely that this also aids flow of particles around one another during pressing. The temperature is not high enough to cause any significant reaction of the SrCl_2_ and TiO_2_ phases, and both are observable as crystalline by XRD after CS (Fig. 1c). The applied pressure ensures that as the water leaves the pellet and that compaction is still achieved. This is observed as a further drop in the applied load upon cooling, to around 50% of the original value. At the end of the pressing, a pellet of the strontium titanate starting powder is formed, with small quantities of crystalline strontium chloride and titanium dioxide surrounding each grain.

**Fig. 3:**
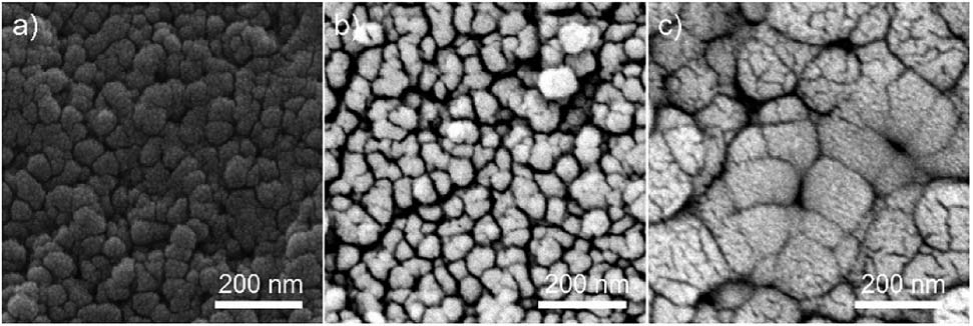
(a) Secondary and (b) backscattered electron imaging of the native surface of a nanopowder sample post heated-pressing. There is a significant secondary phase in (b) surrounding each grain which is no present in (c), the same sample after the secondary heating step, which also shows grain growth due to heating.

SEM of a nanopowder sample after CS but before heating is shown in Fig. 3, and indicates the degree of densification directly after pressing (Fig. 3a). Backscattered electron imaging was also used to show compositional differences in the post press and post second heat samples (Fig. 3b and c respectively). Fig. 3b displays darker areas around the grain boundaries which could be attributed to regions containing a higher quantity of chloride ions than the bulk powder. These darker regions disappear after heating as shown in Fig. 3c, which also indicates that some grain growth has occurred. This grain growth is aided by the surrounding reacting species. The post-press heating step is a vital part of the procedure which promotes the reaction of the strontium chloride and titanium dioxide phases, producing a nanophase of SrTiO_3_ around the bulk powder at 950 °C which fill the empty space surrounding each particle, resulting in a dense, single-phase ceramic. The chemical reaction can be described as follows:SrCl_2_ + TiO_2_ + H_2_O → SrTiO_3_ + 2HCl

Although HCl is formed during this reaction, the SrCl_2_ phase forms only a small fraction of the total mass of the pellet, making the quantity produced negligible.

It is important to make a distinction here between hydrothermal CS and the process here. In hydrothermal CS, the barium hydroxide and titanium dioxide do not form crystalline secondary phases after the hot-pressing step. Instead they react to form an amorphous glass which is then precipitated though TLK growth upon further heating.^9^ This type of amorphous-to-crystalline process is also the main driver for more standard ceramic cold sintering routes. The process presented here is distinct from this, with the intermediate phases reacting at a later stage in the process. This difference is key as it removes the requirement for pristine crystalline surfaces, enabling the use of any size or quality of starting powder. In reactive intermediate CS the titanium dioxide nanoparticles added as part of the CS solution will act as nucleation sites during heating, replicating the reaction observed in the deep eutectic solvent synthesis on the nanoscale around each grain. This means that the starting powder plays no active role in the CS. This is in contrast to other CS processes where the glassy phase precipitates onto the starting powder, giving the reactive intermediate method a greater degree of flexibility.

**Fig. 4:**
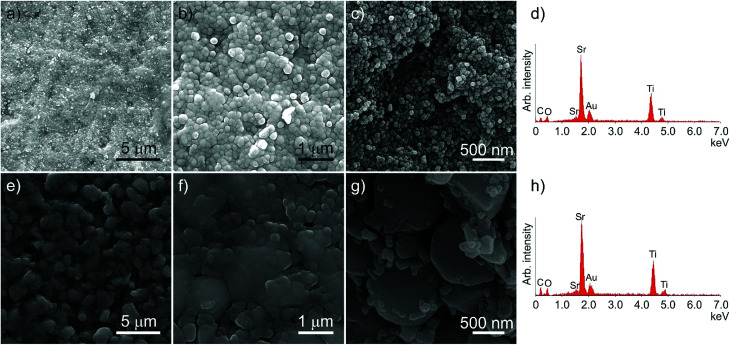
SEM micrographs and corresponding EDXA spectra of the cold sintered nano (a–d) and micron-scale solid state (e–h) ceramics. (a) wide-scale nanopowder native ceramic surface; (b) higher resolution nanopowder native ceramic surface; (c) fracture surface of the nanopowder ceramic; (d) EDXA of area shown in (b), with no chloride ions present; (e) wide-scale solid state micron-scale native ceramic surface; (f) higher resolution solid state micron-scale native ceramic surface; (g) fracture surface of the solid state micron-scale powder ceramic; (h) EDXA of area shown in (f), also with no chloride ions present. Gold is present as the coating material.

SEM was used to examine the microstructure of the sintered ceramics, as shown in Fig. 4. The native surface of the nanopowder ceramic (Fig. 4a and b) reveals a microstructure, commensurate with the high densities obtained using the Archimedes method. The fracture surface (Fig. 4c) indicates that the constituent particles form a single size population (47 ± 12 nm), with densification arising from the ability of the hydrated powder to flow and rearrange into a fully dense ceramic during the CS step, and with any voids latterly filled by the TiO_2_/SrCl_2_ microreaction. EDXA (Fig. 4d) indicated no detectable levels of chlorine in the ceramic after processing. Interestingly, the particle size on the native surface (Fig. 4b) appears larger than the fracture surface. The low melting point of SrCl_2_ means that there is likely to be some liquid phase present during the post-press heating stage. The internal grain structure will constrain these regions, keeping the internal particle size small. The surface is not constrained, and so any liquid phases which form have the opportunity to spread further across the surface resulting in marginally larger grains.

Fig. 4e and f show the native surface of the ceramic produced using solid state micron-scale powder, again in agreement with the high densities attained. The fracture surface (Fig. 4g) shows a bimodal distribution of particles, the larger being the original bulk powder (average size 1.32 ± 0.51 μm, in agreement within errors with the particle size of the raw solid state powder), and the smaller (126 ± 69 nm) originating from the recrystallisation of the materials added during CS.

EDXA of the solid state powder ceramic (Fig. 4h) indicates that, as with the nanopowder ceramic, there are no detectable levels of chlorine left in the ceramic after the post-press heating. This is in contrast to the ceramic examined before the heating step (Fig. S4†) showing high levels of chorine present in EDXA.

**Fig. 5:**
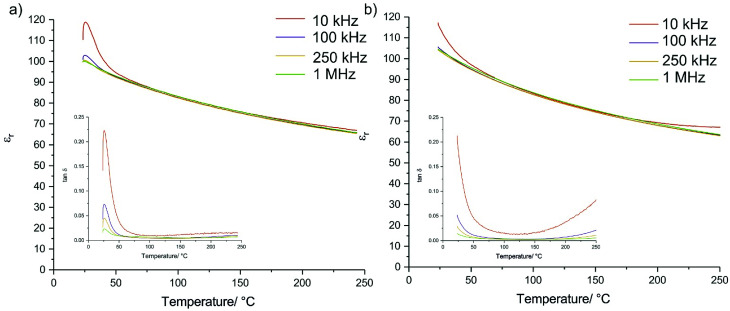
Relative permittivity and loss tangent (inset) for (a) a nanopowder CS ceramic and (b) a solid state powder CS ceramic. A small amount of variation with frequency is observed.

Relative permittivity from room temperature to 250 °C was recorded (Fig. 5), and found to follow similar trends with temperature as conventionally sintered SrTiO_3_ ceramics, albeit with lower relative values (Fig. S5†). The samples prepared using CS show some frequency dependency at room temperatures which indicates some electrical differences between the conventionally and CS samples. This is unsurprising given the difference in the sintering pathway, and so although the sample is compositionally homogenous, further investigation into the exact nature of the electronic properties is required. The frequency dependence is also observed in the loss tangent, although in both cases the values of tan *δ* approach those of conventionally sintered samples (Fig. S5†) above 75 °C. The loss tangents of the CS samples also generally show better stability at the higher end of the measured temperature range.

The high solubility of chloride salts means that the precise concentration of the starting solution can be much higher in molarity than the hydroxide route. Starting with an aqueous solution means that the SrCl_2_ will be homogenously distributed around each particle of SrTiO_3_, irrespective of size. This opens the door for a greatly increased range of materials which can be cold sintered, as not all materials can be easily made or obtained as nanoparticles; this method can be used with solid state powders. Previous work has been entirely dependent on a nanoparticulate starting powder, which are often expensive, difficult to obtain, or unscalable, something which becomes particularly prominent when considering fractionally doped compositions (*e.g.* the n-type thermoelectric material La_0.1_Sr_0.83_Dy_0.07_TiO_3_)^23^ which cannot be easily obtained commercially on the nanoscale or otherwise. This is due to the mechanism by which the amorphous in-filling phase is recrystallized (TLK growth) which requires pristine surfaces upon which solute ions can deposit. In intermediate phase reactive CS, the in-filling phase is reacting with itself rather than the starting powder, so the requirement for a pristine crystal surface is removed. This makes the process applicable across a much wider range of powder compositions and sizes, and also gives the opportunity to make multiphase materials, which would be inaccessible using TLK-based CS.

## Conclusions

In summary, we have presented a cold sintering process which uses intermediate phases which react to create a nano in-fill phase to densify the green body. We have demonstrated this as a method for sintering SrTiO_3_ at 950 °C from feedstocks which include micron as well as nanosized particulates. The methodology creates micro-reactions around each particle during the post-press heating step where the reaction mirrors the synthetic pathway followed by the formation of SrTiO_3_ by deep eutectic solvent synthesis. The wide availability of soluble chlorides, coupled with the added flexibility in particle size which this method can accommodate, offers a highly adaptable route to create fully dense ceramics at reduced temperatures. Additionally, different micro-reactions could be selected making this processing technique highly adaptable for making fully dense ceramic/ceramic composites. This method is distinct from other CS processes as it follows a different mechanism for the formation of the infilling phase. As this is a reaction rather than a TLK precipitation, the requirement for pristine nanoscale surfaces is removed, creating the opportunity to cold sinter micron-scale materials *i.e.* those created using standard solid-state processing, as demonstrated here. This, and the wider availability of soluble metal chlorides and reactions of a similar style, make this a step towards a general cold sintering strategy for complex functional oxides.

## Conflicts of interest

There are no conflicts to declare.

## Supplementary Material

RA-008-C8RA03072C-s001
