# Supplementary material for: Reactive intermediate phase cold sintering in strontium titanate
Source: RSC Adv. 2018 Jun 4;8(36):20372–8. doi: 10.1039/c8ra03072c (PMC9080801; doi:10.1039/c8ra03072c)
Supplement: RA-008-C8RA03072C-s001 [file RA-008-C8RA03072C-s001.pdf]

Electronic Supplementary Material (ESI) for RSC Advances.
This journal is © The Royal Society of Chemistry 2018

Supporting Information

Title: Reactive intermediate phase cold sintering in strontium titanate

Rebecca Boston, Jing Guo, Shuichi Funahashi, Amanda L. Baker, Ian M. Reaney and Clive. A. Randall*

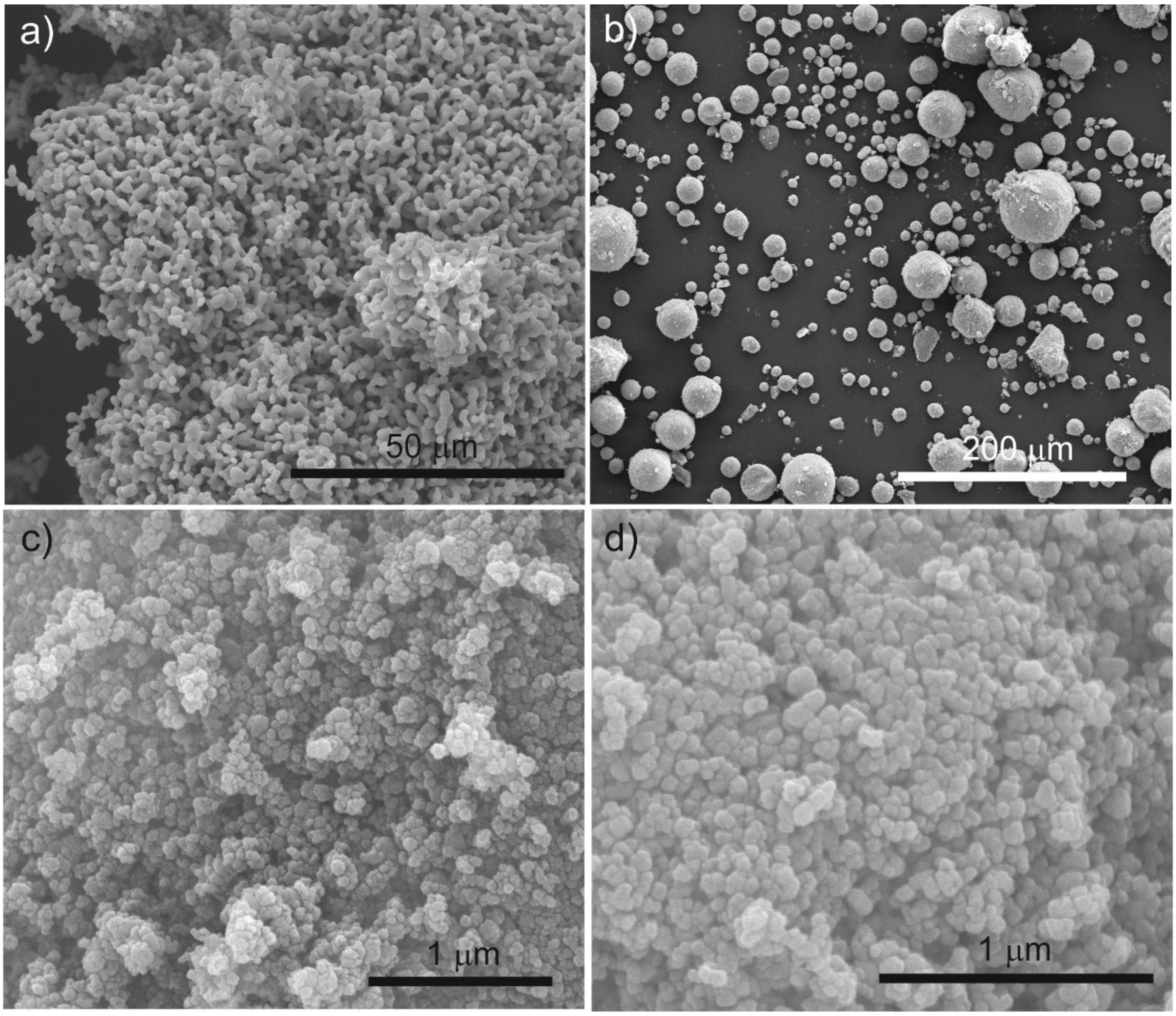

Figure S1. SEM micrographs of a) the in-house-synthesised solid state $SrTiO_3$ b) the as-received $SrTiO_3$ nanopowder which was agglomerated into large particles, c) a higher-magnification image of the $SrTiO_3$ nanopowder showing the crystallite size, and d) the as-received TiO_2 powder.

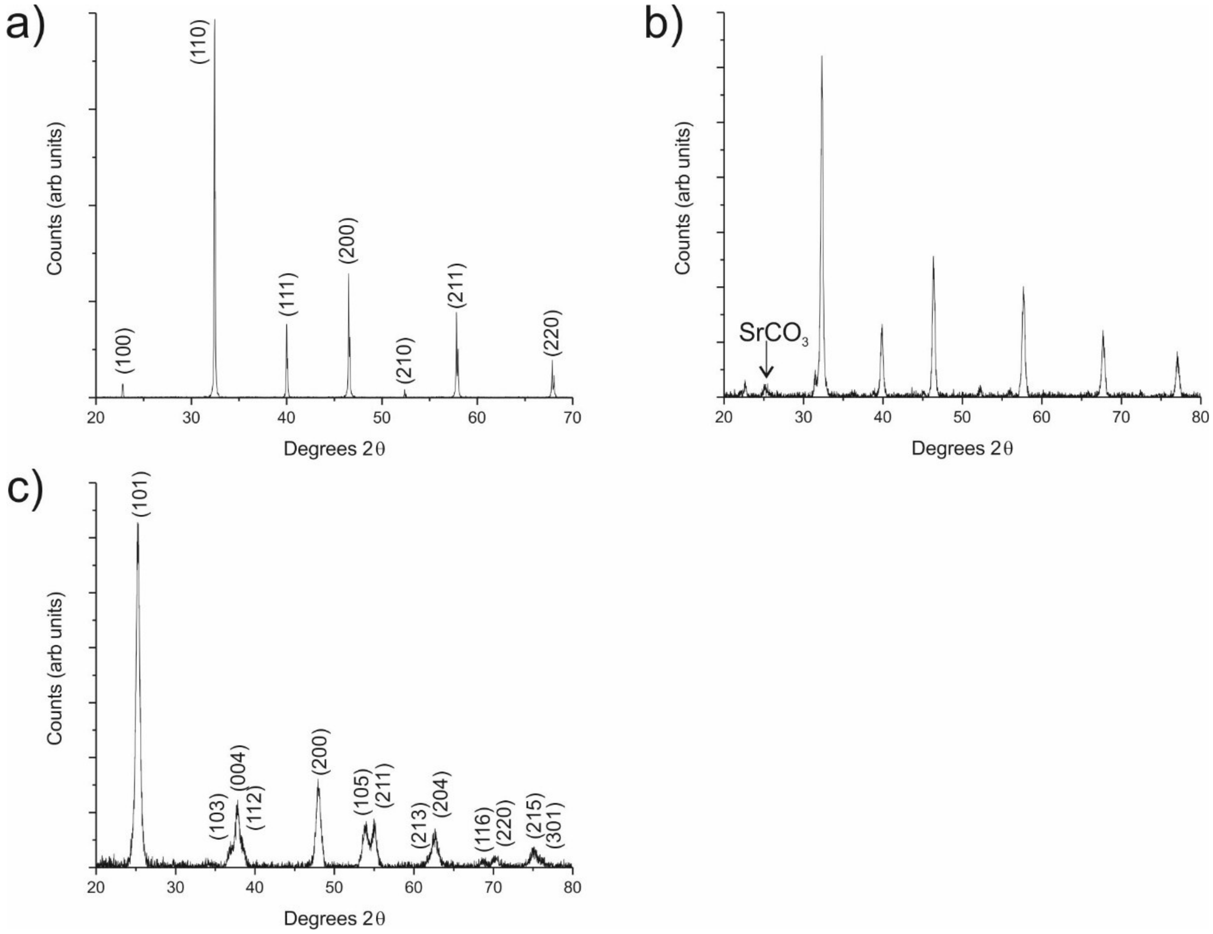

Figure S2. XRD patterns of a) the in house-produced solid state $SrTiO_3$ starting powder indexed to a cubic cell, b) the as-received nano-$SrTiO_3$ powder which contains some $SrCO_3$ phase, and c) the as- received nano TiO_2 powder which showed significant peak broadening due to the particle size.

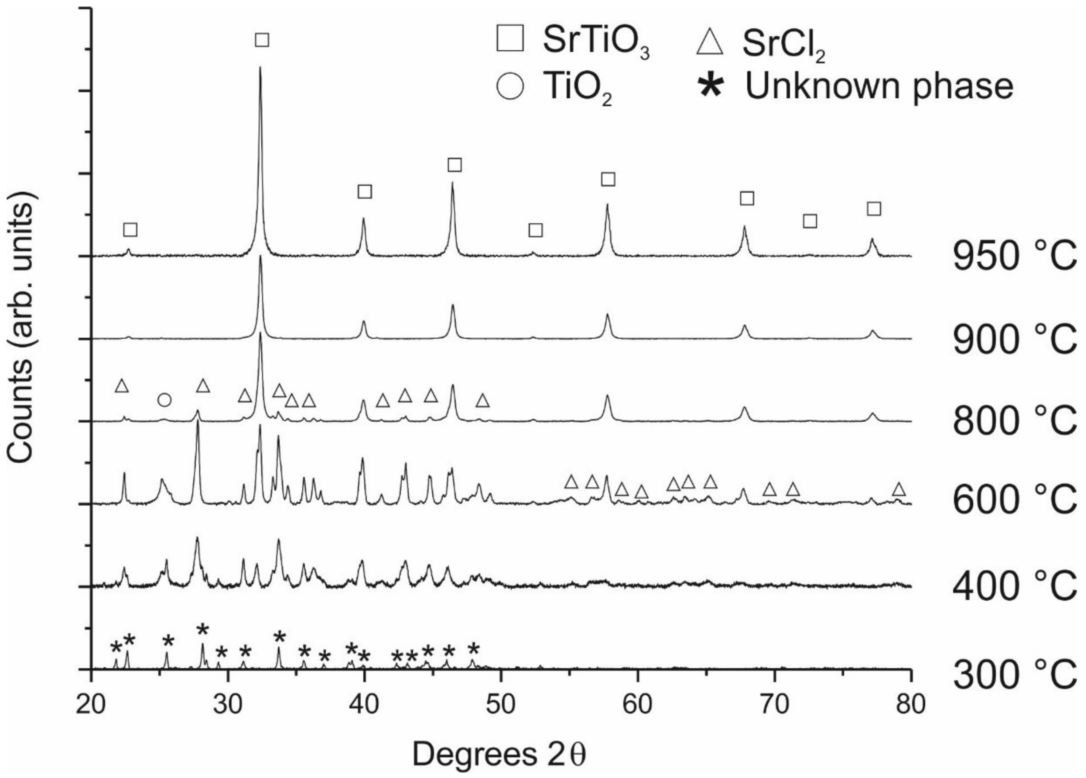

Figure S3. XRD of deep eutectic solvent-synthesised powders calcined at different temperatures. An unknown phase is present below 400 °C, possibly a titanium-organic complex. By 400 °C, this is reacted to form strontium chloride and titanium dioxide, which persist until 900 °C. At 600 °C, the first indication of $SrTiO_3$ is observed. By 950 °C, the intermediate phases have reacted to produce phase pure strontium titanate.

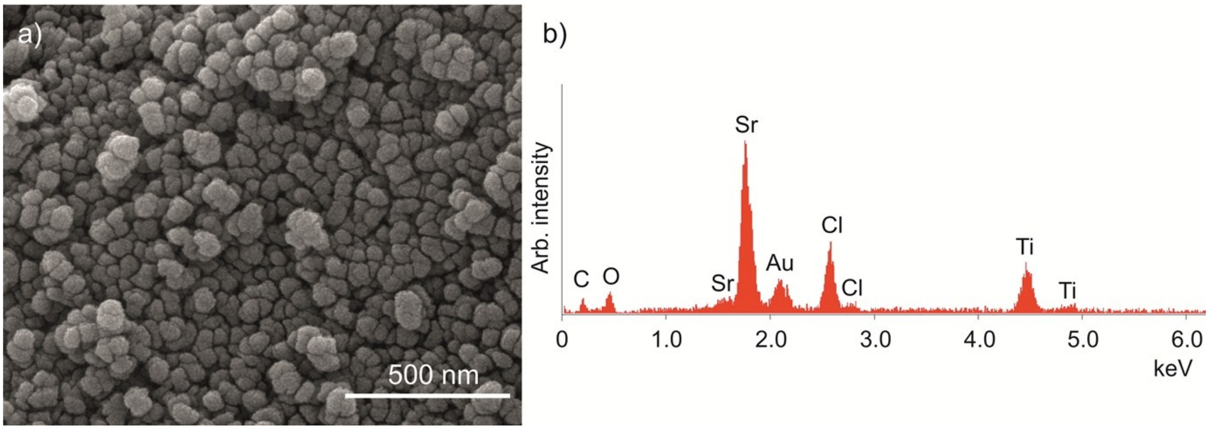

Figure S4. a) SEM and b) corresponding EDXA of a nanopowder ceramic which has been undergone the heated pressing step but has not been heated to 950 °C. The EDXA demonstrates the presence of chlorine (as crystalline strontium chloride) which is later lost in the post-press heating step.

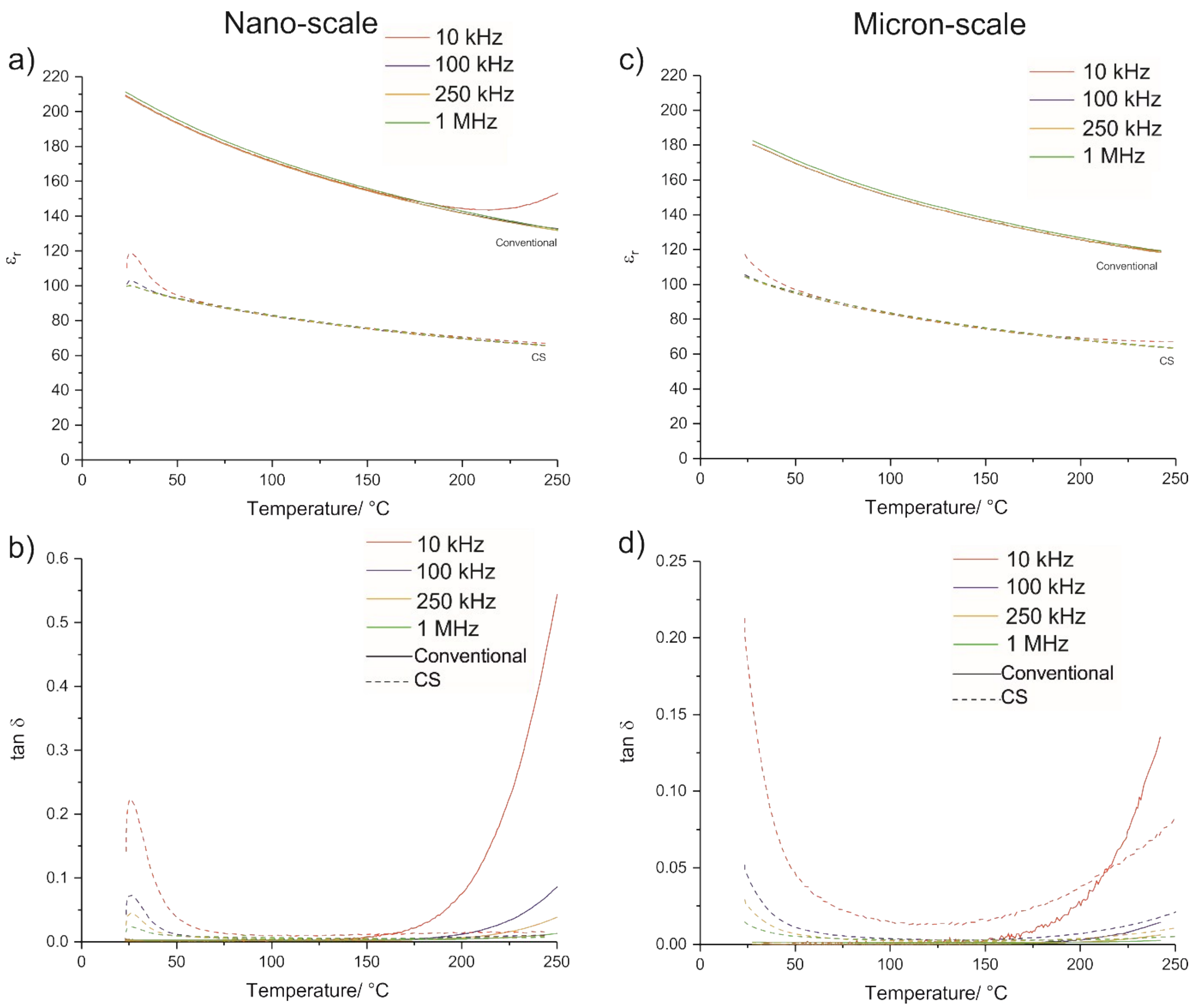

Figure S5. Relative permittivity and loss tangent data for a) and b) nanoscale samples sintered using CS and conventional processes (1400 °C, 6h) and c) and d) microscale samples.
